# Validity and Reproducibility of a Revised Semi-quantitative Food Frequency Questionnaire (SQFFQ) for Women of Age-group 12-44 Years in Chengdu

**Published:** 2015-03

**Authors:** Ying Tang, Ying Liu, Liangzhi Xu, Yujian Jia, Dan Shan, Wenjuan Li, Xin Pan, Deying Kang, Chengyu Huang, Xiaosong Li, Jing Zhang, Ying Hu, Lingli Konglin, Jing Zhuang

**Affiliations:** ^1^Department of Ultrasonography, West China Women's and Children's Hospital of Sichuan University, China; ^2^Department of Obstetrics and Gynecology, West China Women's and Children's Hospital of Sichuan University, China; ^3^Mother and Baby Center, School of Medicine and Public Health, University of Newcastle, Australia; ^4^Department of Evidence-based Medicine and Clinical Epidemiology, West China Hospital, Sichuan University, China; ^5^Department of Nutrition and Food Safety, Sichuan University, China; ^6^West China School of Public Health, Sichuan University, China

**Keywords:** Female, Nutrition assessment, Reliability, Semi-quantitative food frequency questionnaire, Validity, China

## Abstract

To find a credible nutritional screening tool for evaluating relationship between nutritional status and diseases in Chengdu female residents, the reliability and validity of a revised semi-quantitative food frequency questionnaire (SQFFQ) were tested. The validity was assessed by comparing the SQFFQ with the ‘standard’ method of 3 days’ dietary recall, and the reliability was assessed by comparing the first SQFFQ with the second SQFFQ at 4 weeks interval. Correlation analysis showed that, for reliability, the average correlation coefficient (CC) of 22 kinds of nutrients was 0.66 and reduced to 0.60 after adjusting for energy; the average of intra-class correlation coefficients (ICC) was 0.65. For validity, the average CC was 0.35 and remained stable after adjusting for CC of energy or nutrients. Validity of 17 nutrients in SQFFQ survey had correlation with result of 3 days’ dietary recall. The results showed that the revised SQFFQ can be used for investigating the role of nutrients in development of disease in Chengdu female residents.

## INTRODUCTION

The semi-quantitative food frequency questionnaire (SQFFQ) is the most commonly-used tool for nutritional epidemiological dietary survey in recent years. Considering the differences in geography, culture, and dietary habits in different people, a different SQFFQ is used or an appropriate modification of the existing questionnaire, and re-evaluation of its reliability and validity are needed before the application of SQFFQ.

Females in the age-group of 12-44 years were under high risk of metabolism-related menstrual disorders. Take polycystic ovary syndrome (PCOS), for example, which is characterized by its excessive androgen secretion and chronic anovulation or oligo-ovulation and has great influence on the metabolism of sugar, fat, and protein. Recent studies found that dietary glycaemic index and increased energy intake were associated with less favourable anthropometric and metabolic profile in PCOS women (1,2). Experts in obstetrics and gynaecology, endocrinology, nutrition, and epidemiology all paid great attention to this disease. So, studies for understanding the relationship between metabolism-related diseases and dietary pattern are needed to be carried out in females in the age-group of 12-44 years. Currently, there is still a lack of epidemiological data on the Chinese people. To understand the relationship between metabolism-related diseases with sugar, fat, protein, and other nutrients, we need an effective tool, such as the food frequency questionnaire. The questionnaire should be able to reflect the real pattern of food intakes, especially sugar, fat, and protein and must be repeatable. However, currently in China, we do not have a suitable food frequency questionnaire for 12-44 years old non-pregnant women. A specific measurement tool for studying the relationship between disease and nutritional intake in women of this age-group is greatly needed. So, in our study, we tested the reliability and validity of an appropriately-modified semi-quantitative food frequency questionnaire (SQFFQ) in 12-44 years old women. The SQFFQ was first used in 35-55 years old healthy adults (both males and females) in Chongqing (3), a place geographically adjacent to Sichuan province.

## MATERIALS AND METHODS

### Subjects and study plan

Chengdu has 9 districts; each district has several neighbourhood offices. According to the Chengdu demographic statistical data (4), 521.52 million females live in Chengdu, each district having roughly the same number of female residents. Female students, women living in the community, and women who work at a special place have more stable living habits, which can reflect the characteristics of the diet in Chengdu women.

According to the nutritional epidemiological theory and epidemiological data in domestic studies (5–9), when the sample-size is about 100-200, it is able to reflect the dietary status of the target population and avoid the problem of inadequate sample-size due to abortion. So, in our study, we attempted to extract 100-200 females aged 12-44 years. Calculation of the exact number of people at different age-groups was based on the census of the Yearbook of Sichuan Province in 2004 (4). We used a random number table for sampling the subjects. Our subjects were selected in Chengdu which consisted of 4 districts; students in six dormitories for females from each grade of a boarding high school (first grade to the third grade of junior, first grade to the third grade of senior); students from two units of dormitory building for females in an urban university; residents from one unit in a residential community; and employees in a department, who lived in two residential communities. All of the subjects were long-term residents in Chengdu and were willing to participate in the study. We excluded women suffering from serious diseases: heart disease, cerebrovascular disease, liver and kidney diseases, cancer, diabetes, organ transplantation, and nutrition-related diseases. Women at pregnancy or lactation were also not included.

### Formulation of a semi-quantitative food frequency questionnaire

In the past, Chengdu and Chongqing both belonged to Sichuan province (Chongqing was part of Sichuan province but now separated as a municipality); people living in these two places share the same properties of food and living habits. So, in our survey, a semi-quantitative food frequency questionnaire, which was first administered and tested for its reliability and validity in Chongqing at 2003, was used. We made some appropriate changes according to the diet and the types of food in Chengdu. The modified questionnaire included 18 categories and 120 kinds of food. The food intake frequencies, together with the average consumption (monthly, weekly, daily), were investigated (average consumption of food was quantitated by multiples of the reference food weight; reference food weight was defined as the average food weight of the most commonly-eaten unit in grammes). Questionnaire with incomplete information or logical error due to logic check over 60% was also excluded in case of bias. Coloured atlases of reference food were used in order to help better understanding of food serving.

### Process of research

This is a cross-sectional study. All subjects received the second questionnaire after 4 weeks interval after the completion of the first food frequency questionnaire.

We took the 24-hour dietary recall method in three days as the ‘standard’ method for evaluating the validity of SQFFQ. The SQFFQ questions were given to the subjects at first, and then in the same week, we gave them 24-hour dietary recall questionnaire. SQFFQ was designed in a multiple-choice response format. The investigation was done by using the combination of face-to-face interview, together with telephone inquiries recommended by Rockett *et al*. (10). Choice of survey sites and method was based on the subject's will. If we could not do the interview face-to-face, telephone interview was applied as a supplemental method with detailed records. Sets of questionnaire were retrieved in the day of investigation.

The open-response format was applied in 24-hour dietary recall questionnaire, all diets from Thursday to Saturday were recorded, and dietary changes in the weekend were also considered. The records included types and weights of digested food throughout the days of investigation at home or outside (restaurants or canteens). When the students could not recall, we asked their teachers. Family members and colleagues also helped the subjects recall, they provided the actual food consumption data and types of food. The survey results were taken back at the fourth day. If the questionnaire could not be taken back, telephone contacts with the subjects were made one week later, and the questionnaire was recycled.

Quality control of the investigation was maintained by the inspectors. We ensured that all of the subjects got an integral and clear SQFFQ. Investigators received a one-day on-site training; after passing in the examination, they could take part in our survey. In our study, we finally included 4 investigators. Pre-investigation was carried out before the formal investigation so that we could give the investigators more suggestions on communication and consuming time. Data in each questionnaire were double-checked by inspectors before putting into the database. If inconsistencies were found, we would call the subjects immediately and re-record the questionnaire at the presence of both investigators and the inspectors. Two independent programmers put the data into our database after verification.

### Statistical analysis

The calculation of nutrients intake was based on the Chinese Food Composition Table (11). We used Nutrition Calculator (version 1.6), which is designed by the China Nutrition and Food Safety, together with Feihua Healthnet, to analyze the nutrients intake. The weight of daily food intake was calculated using the first and the second SQFFQ obtained (Food weight=Frequency of food intake × Standard weight of each kind of food × Multiples of the standard weight in daily intake). The data of daily food intake from 24 hours dietary recall questionnaire were calculated (Daily food intake=1/3 of total food intake in 3 days).

### Calculation of condiments

For the students eating in the school, the school cafeteria provided us the type and amount of oil (12); for subjects eating out, the daily intake of oil was based on the average of oil intake in Sichuan province; for subjects eating at home, edible oil was calculated though investigating the type and amount of meals consumed per person per day.

Different methods of calculation were applied depending on different types of data. Normal distribution of data was described using the mean and standard deviation; the skewed distribution of data was described using the median and interquartile range and was converted to data of normal distribution.

### Test of reliability and validity

Pearson's correlation coefficients of the first and second SQFFQ (reliability) were calculated, with the Pearson's correlation coefficients of the first SQFFQ with the 24-hour dietary recalls method (validity). The residual method was applied for energy adjustment. To correct and eliminate inter-individual and intra-individual variation, intra-class correlation coefficient (ICC) was calculated using the formula: 
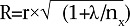
 (r=Pearson's correlation coefficient after logarithmic conversion, x=days of repeating time; in this study, x=3, λ=ratio between intra-individual variation and variation in different individuals; the calculation of λ was based on expert suggestion on variance model) (13,14). When the correlation coefficients of nutrients intake was statistically significant (p<0.05), it was considered reliable. Statistical software SPSS (version 13.0) and Excel were used for data analysis.

### Ethical clearance

Informed consent from all participants before entry into the study was obtained. This epidemiological survey was approved by the local ethics committee of Institutional Review Board (IRB), and the protocol was executed in accordance with the ethical principles stated in the Declaration of Helsinki. Each participant granted their participation in the study by verbal or written informed consent.

## RESULTS

### Characteristics of the subjects

One hundred and forty-seven women were recruited in this study. Thirty-seven were excluded because of quitting (8 of the subjects), acute disease (2 of the subjects), incomplete information or logical error in questionnaire over 60% (11 of the subjects), and lost to follow-up (6 of the subjects). Finally, 110 women were included in this study. The age distribution of the subjects was in accordance with that of the female population in Chengdu (4) (Table 1).

The result of follow-up in our study was real and credible. In the gap between recruited participants and studied participants, 11 of the subjects had been excluded because of incomplete information or logical error due to logic check over 60% in questionnaire; so, the actual rate of dropout was 18%, and it is acceptable according to the theory of clinical epidemiology which requires dropout rate below 20%. Actually, as the conclusion of our result suggests that the proposed SQFFQ was reliable and valid for further study, there is attrition bias which could decrease reliability and validity; the 18% dropout would strengthen this result.

**Table 1.** Characteristics of the subjects in the survey of SQFFQ reliability and validity

Characteristics	Number of participants	Percentage
Age (completed years)		
12-17	10	9
18-29	45	41
30-39	44	40
40-44	11	10
Educational background		
≥College	59	53
Middle school	36	33
Elementary school	15	14
Career		
Student	53	48
Employed	51	46
Unemployed	6	6

### Characteristics of nutrition intake in subjects

The daily intakes of vitamin A and calcium were lower than the recommended dose of the National Institute of Nutrition (700 μg/day, 800 g/day respectively). The daily intakes of vitamin E, niacin, phosphorus, and manganese were higher than the recommended dose by Chinese Nutrition Society (14 mg/day, 13 μg/day, 700 mg/day, and 3.5 mg/day respectively) (Table 2).

### Reliability of SQFFQ

Table 2 shows the average of the first and the second SQFFQ with monthly intervals and the 3-day recall method. After logarithmic transformation, the Pearson's correlation coefficients of the first and the second SQFFQ were between 0.50 and 0.75, with an average of 0.66. The average ICC was 0.65. All of the 22 nutrients were positively correlated (Table 3), which indicated a good reliability of this questionnaire .

### Validity of SQFFQ

Pearson's correlation coefficients of SQFFQ and three days of 24-hour dietary recalls method were between 0.12 and 0.69, with the average of 0.35; after adjusting for energy intake and correlation coefficients, the average became 0.32. In 22 kinds of nutrients, the intakes of energy, protein, fat, carbohydrates, dietary fibre, cholesterol, niacin, sodium, and manganese showed a good authenticity. The p value for the correlation coefficients of phosphorus, magnesium, selenium, copper, and niacin were above 0.05, indicating that the intakes of these five kinds of nutrients of SQFFQ were irrelevant with the results of the 3-day recall method (Table 4).

## DISCUSSION

In previous studies in Chongqing, the researchers had established an SQFFQ, which was targeted on 35-55 years old people, with no difference in gender. However, as we all know people of different sexes and different ages have different dietary preferences which would lead to diversity, a specific measurement tool to study the pathogenesis of diet in female adolescents and women of childbearing age must be established. In our country, however, the reliability and validity tests for SQFFQs were only performed in pregnant women, 40 to 70 years old females, and middle-aged people (3,15–18). In view of this, we modified the Chongqing SQFFQ and retested its reliability in this study. Our results showed that the Pearson's correlation coefficients of the 22 nutrients were between 0.50 and 0.75, with an average of 0.66. In addition, we used the 24-hour dietary recall questionnaire of 3 days as the standard method, which provided detailed assessment of the daily nutrients intake and also efficiently helped detect the validity of SQFFQ. The Pearson's correlation coefficients were between 0.12 and 0.69, with an average of 0.35. The results were similar to other studies at home and abroad (19–21), indicating that the modified Chongqing SQFFQ can be applied to the 12-44 years old women in Chengdu.

### Reliability of SQFFQ

When we calculate the correlation coefficients after logarithmic conversion, the Pearson's correlation coefficients of 22 kinds of nutrients were between 0.5 and 0.75, with an average of 0.66, the ICCs were between 0.45 and 0.75, with an average of 0.65. These results were similar to most of the SQFFQ studies, in which the correlation coefficients were between 0.4 and 0.9 (19–21), indicating the good reproducibility in this SQFFQ study. In addition, the correlation coefficient of Chongqing SQFFQ was 0.67 (18), which is very close to the average correlation coefficient in our study, indicating the consistency of nutrients intake by females between these two cities. We can come to the conclusion that the modified Chongqing questionnaire is suitable for our study in Chengdu females.

**Table 2.** Median of SQFFQ1, SQFFQ2, and 3-day dietary recall method

Nutrients (N=110)	SQFFQ1 Median (25%-75%)	SQFFQ2 Median (25%-75%)	3-day recall method Median (25%-75%)
Energy (kal/day)	2,083.1 (1,590.3-2,552.5)	2,193.44 (1,510.5-2,687.8)	1,928.30 (1,587.8-2378.2)
Protein (g/day)	80.4 (52.3-99.5)	85.5 (50.5-103.1)	66.78 (51.3-81.9)
Fat (g/day)	92.8 (66.1-105.4)	100.70 (63.9-118.2)	85.43 (74.1-94.1)
Carbohydrates (g/day)	242.0 (166.8-315.2)	247.6 (155.5-321.2)	224.62 (177.2-266.9)
Dietary fibre (g/day)	12.66 (6.6-15.3)	12.9 (6.5-15.8)	10.34 (5.6-13.1)
Cholesterol (mg/day)	276.2 (157.3-342.8)	326.12 (155-381.3)	348.9 (188.6-480.3)
Vitamin A (µg/day)	744.7 (316.8-925)	976.91 (287.5-917.8)	473.78 (281.83-566.42)
Thiamine (mg/day)	1.3 (0.8-1.6)	1.4 (0.8-1.7)	5.4 (0.7-1.1)
Riboflavin (mg/day)	1.0 (0.6-1.2)	1.20 (0.6-1.4)	0.8 (0.6-0.9)
Niacin (µg/day)	17.5 (11.12-22.3)	18.7 (10.3-22.4)	14.00 (10.6-16.7)
Vitamin C (mg/day)	109.3 (55-152.3)	112.2 (47-145)	58.92 (41.3-69.7)
Vitamin E (mg/day)	26.4 (19.1-31.4)	26.6 (18.1-31.9)	30.3 (25.6-34.3)
Calcium (mg/day)	506.8 (277.5-637.8)	533.0 (297.3-611.0)	408.8 (285.6-493.3)
Phosphorus (mg/day)	1191.8 (765.5-1391.3)	1,223.2 (716-1,522.8)	977.3 (704.8-1,053.0)
Potassium (mg/day)	2241.8 (1,371.3-2,778.5)	2,340.03 (1,260-2,947)	1,640.7 (1,306.4-1,915.2)
Magnesium (mg/day)	308.9 (196.0-378.3)	324.4 (188.0-401.0)	242.3 (181.4-293.7)
Iron (mg/day)	24.5 (15.5-27.5)	28.4 (14.8-32.5)	19.5 (13.4-21.2)
Zinc (mg/day)	12.0 (8.4-15.0)	13.1 (7.7-16.1)	10.9 (8.5-13.1)
Selenium (µg/day)	45.5 (30.8-55.1)	49.8 (29.5-59.1)	40.0 (31.2-48.8)
Copper (mg/day)	2.3 (1.5-2.7)	2.4 (1.3-2.9)	1.8 (1.3-2.1)
Manganese (mg/day)	4.9 (3.5-6.1)	5.0 (3.1-6.0)	4.9 (3.2-6.2)

### Validity of SQFFQ

Some domestic and foreign studies found that SQFFQ can effectively reflect the nutrients intake status of the target population and is an economical and reliable method in the large-sample population surveys (22,23). However, the method relies on the memories of subjects; therefore, its validity is of great significance for the judgement of the results. Currently, there is no accepted ‘standard’ to test the accuracy of the dietary survey method. Some scholars suggested the 24-hour dietary recall method as a major standard way because of its no limitation on age (24). The operation of this method is comparatively simple, saving manpower and resources. It is especially suitable for the survey with a large number of subjects and where participants lack enthusiasm and education.

**Table 3.** Correlation coefficients of SQFFQ in reliability study (SQFFQ1 vs SQFFQ2)

Nutrients (N=110)		Pearson's correlation coefficient		ICC
		After logarithmic transformation	After adjusting for energy intake	
Total energy	Energy*	0.69	-	0.68
Three major nutrients	Protein*	0.65	0.58	0.64
	Fat*	0.63	0.53	0.61
	Carbohydrate*	0.70	0.56	0.70
Dietary fibre	Dietary fibre*	0.75	0.72	0.86
Cholesterol	Cholesterol*	0.60	0.57	0.56
Vitamins	Vitamin A*	0.61	0.55	0.58
	Thiamine*	0.59	0.42	0.52
	Riboflavin*	0.66	0.65	0.63
	Niacin*	0.63	0.51	0.60
	Vitamin C*	0.73	0.72	0.72
	Vitamin E*	0.75	0.75	0.73
Elementary elements	Calcium*	0.71	0.67	0.71
	Phosphorus*	0.68	0.63	0.67
	Potassium*	0.68	0.62	0.66
	Sodium*	0.69	0.65	0.67
	Magnesium*	0.62	0.52	0.60
Trace elements	Iron*	0.68	0.70	0.65
	Zinc*	0.62	0.45	0.61
	Selenium*	0.50	0.43	0.45
	Copper*	0.72	0.67	0.70
	Manganese*	0.62	0.64	0.62

*p<0.05

ICC=Intra-class correlation coefficient

In this study, 24-hour dietary recalls of three consecutive days were used. Using the combination of face-to-face and telephone interviews is a time-saving method and can avoid traffic problems in advantage. Telephone interviews without appointment can prevent the subjects from preparing the standard answers (25,26). The review time of 24-hour dietary recalls method from investigation is shorter than in other methods, and the likelihood of memory errors is relatively low; so, we take it as the method for validity evaluation. In addition, our study used three consecutive days of 24-hour dietary recalls; weekdays and weekends were all included, which can truly reflect the nutrition intake of the Chengdu females.

In our study, we compared SQFFQ with the 3-day dietary recall record. We found that the intakes of 18 of the 22 kinds of nutrients were higher when using SQFFQ; the intakes of cholesterol, vitamin A, and vitamin E were lower; and intake of manganese was consistent with the other sets of questionnaire. The average overestimation was 24% higher in SQFFQ. The degree of overestimation was similar to the Chongqing SQFFQ weighing method and also similar to other studies (17,27,28). The reason of overestimation comes from the long-term memories of people when finishing this questionnaire; the opinion of ‘do not miss’ always makes the subjects tend to overestimate the amount of food intake (13,29). However, the patients and controls all have the overestimation tendency; so, the differences between the two do not change. Therefore, the results of the study are not affected. In addition, some scholars compared dietary records and dietary recalls method in the same group of people; the results showed no statistical difference; using the recall method can reflect the usual dietary intake (30,31).

**Table 4.** Correlation coefficients of SQFFQ in validity study (SQFFQ1 vs 3-day recall method)

Nutrients (N=110)		Pearson's correlation coefficients		ICC
		After logarithmic transformation	After adjusting for energy intake	
Total energy	Energy*	0.64	-	0.60
Three major nutrients	Protein*	0.60	0.47	0.57
	Fat*	0.58	0.40	0.58
	Carbohydrate*	0.49	0.41	0.47
Dietary fibre	Dietary fibre*	0.69	0.70	0.69
Cholesterol	Cholesterol*	0.41	0.29	0.39
Vitamins	Vitamin A*	0.33	0.38	0.33
	Thiamine*	0.24	0.19	0.21
	Riboflavin*	0.27	0.20	0.24
	Niacin	0.52	0.46	0.49
	Vitamin C*	0.38	0.41	0.32
	Vitamin E*	0.29	0.37	0.23
Elementary elements	Calcium*	0.38	0.38	0.36
	Phosphorus	0.15	0.17	0.15
	Potassium*	0.34	0.35	0.32
	Sodium*	0.48	0.39	0.47
	Magnesium	0.14	0.11	0.12
Trace elements	Iron*	0.12	0.15	0.11
	Zinc*	0.16	0.23	0.14
	Selenium	0.15	0.24	0.15
	Copper	0.16	0.25	0.14
	Manganese*	0.43	0.52	0.41

*p<0.05

ICC=Intra-class correlation coefficient

The Pearson's correlation coefficients were between 0.12 and 0.69 after logarithmic transformation, with an average of 0.35. The Pearson's correlation coefficients and ICC of energy, protein, fat, carbohydrates, dietary fibre, cholesterol, niacin, sodium, and manganese were between 0.39 and 0.4, implying the authenticity of these nutrients intake. The correlation coefficient of dietary fibre was 0.69, which was the highest. The results obtained from SQFFQ questionnaire were closely relevant to the 3-day dietary recall method in 12-44 years old women in Chengdu; and it could truly reflect the dietary intake of this age-group. In particular, this SQFFQ questionnaire provided us a more credible assessment of the main source of energy, protein, fat, carbohydrates, energy metabolism, and the health-related dietary fibre intake. These results are consistent with the results when SQFFQ was used in the research of type 2 diabetes, which is also a chronic metabolic disease (27).

In the study of FFQ validity, experts recommended using the corrected correlation coefficient to eliminate the impact of intra-individual and inter-individual variability. In this study, the average Pearson's correlation coefficient after logarithmic transformation was 0.35; the average had an increase of 0.05 after adjusting for correlation coefficients. In the survey conducted in Chongqing, the average had an increase of 0.03 (18). The consistence of these two studies indicated that the modified SQFFQ can reflect the status of Chengdu women's dietary intake of nutrients more accurately.

Some scholars proposed that correction after adjusting for energy should be conducted in nutrition analysis (32) because total energy intake may have an influence on the kinds of nutrients intake or may interfere with the relationship between a special nutrient intake and disease or a special nutrient intake and subjects’ preference. In this study, the Pearson's correlation coefficients of most of the nutrients were lower after adjustment; the average correlation coefficient of validity was 0.34; and the average correlation coefficient of reliability was 0.60. This decrease in validity and reliability after adjustment corresponds to the Chongqing SQFFQ, implying the influence of total energy intake on the single nutrient intake. In the circumstances of a constant total energy intake, increasing the intake of a certain nutrient should be based on choosing the food rich in that nutrient.

### Factors affecting reproducibility and validity

The determination of survey period, number of days for the survey, and the test-retest interval is very important in SQFFQ study. The 24-hour dietary record method can only reflect the eating status in one day and does not reflect the seasonal food intakes and long-term eating habits. In addition, some scholars believed that the difference between the daily diets in one week exists, especially on weekdays and weekends. Taking all these opinions into account, we used 24-hour dietary recalls method three times a week. We selected Sunday and two weekdays to cover the possible differences (33), which may better reflect the eating habits.

The interval of retest reliability was from 1 week to several years in different studies; some foreign studies have shown that the reliability coefficients of nutrients in retest studies were between 0.4 and 0.7 at 1 week to 10 years interval. We have not found the same domestic research as reference. However, taking into account the actual situation of local and future studies and, according to the similar studies undertaken abroad (22,34–36), we determined the intervals for one month in our research. The correlation coefficient obtained in our research is similar to that in other foreign studies, which ensured the viability of using this time interval.

Human memory is influenced by educational background, physical condition, gender, and mood. The subjects’ recall bias is the main factor that affects the results in any investigation. Displaying a food image to the subjects is a good way to help them estimate the weight of food. Estimating the weight of food is always the difficult part in a dietary survey. Borrud tried to apply photos of the food into the estimation of food-size and found that the results with the gravimetric method were pretty close (37). In this study, we used the standard-weight pictures of food (per bowl, dish, etc.) to help the subjects recall, making the estimation of the food more objective and more realistic.

Nutritionists usually recommend to use the existing questionnaire or modify the current questionnaire in an epidemiological survey. For people living in the same places, sharing the same cultural background, and having the same eating habits, using these sets of questionnaire can be a very convenient way (32,33,38). Chongqing and Chengdu are proximal geographically; people of these two places share the same living and eating habits. So, we used the SQFFQ established in Chongqing nutrition examination survey and modified it. Due to the differences between age and gender in these two studies, we need to retest the reliability and validity before using this modified questionnaire. This questionnaire has two parts: frequency question part and open question part. In the frequency question part, the subjects need to determine a few options about the frequency of food intake. In the open question part, subjects need to write the frequency of food without the restrictions of the options. Therefore, women with low level of education and low cognitive ability can do the frequency question part faster with few omissions. In the process of survey, subjects may generate boredom or curiosity, and that may affect the accuracy of SQFFQ.

### Strengths and limitations

This study is based on the actual eating habits of the female residents in Chengdu. We selected 12-44 years old female population according to the Sichuan Yearbook, which makes it more representative of the real status of Chengdu females. This study provided us a reliable tool in the research of the relationship between disease and nutrition intake.

All the investigations were carried out by well-trained and experienced investigators, making most of the interviews processed successfully. In addition, without the restrictions of time and traffic, telephone interviews greatly improved the acceptance and compliance of the subjects. The use of coloured food atlas to help the subjects recall enhanced the accuracy of the questionnaire.

Our study had a few limitations, however. The China Food Composition Table (2002 edition) failed to include all foods in the questionnaire; so, an objective calculation based on that may affect the results of some nutrients intake. Two kinds of geographically-special vegetables are not included in the table; we did the calculation by putting them into the similar kind of vegetables. This may have little influence of the outcome due to the small amount (only 6 people consumed) and very low intake frequency of those two vegetables. Another eight kinds of Chinese food condiments (refined salt, soy sauce, vinegar, chili powder, chili sauce, sugar, pepper, and monosodium glutamate) were used in small amount in the cooking process. The intake of those condiments was closely related to personal preferences. The subjects only made a rough estimation based on the photo of food. That might affect the final estimation of the intake of these nutrients.

### Conclusions

In general, this SQFFQ can reflect the nutrients intake status of Chengdu women with accuracy and reliability; it can reflect important nutritional sources of energy, protein, fat, carbohydrates, and dietary fibre. The SQFFQ can be recognized as an ideal dietary assessment tool in the research of relationship between nutrient intake and disease in the 12-44 years old female residents of Chengdu.

## ACKNOWLEDGEMENTS

This study is sponsored by National Natural Science Foundation of China (Dr. Liangzhi Xu, Principal investigator, Project No. 81270665 and Project No. 41473097), and research projects from Health Department of Sichuan province (Dr. Liangzhi Xu, Principal investigator, Project No. 090289). The authors also thank Li Ning-Xiu for study design; volunteer Hong Pan, Lan Chen, Li Li, Honghao Li, and Jia Xie, for questionnaire survey.

**Conflict of interest:** Authors declare no competing interests.
